# Comment on Cannarella et al. DNA Methylation in Offspring Conceived after Assisted Reproductive Techniques: A Systematic Review and Meta-Analysis. *J. Clin. Med.* 2022, *11*, 5056

**DOI:** 10.3390/jcm11236908

**Published:** 2022-11-23

**Authors:** Bastien Ducreux, Patricia Fauque

**Affiliations:** 1 Université Bourgogne Franche-Comté-Equipe Génétique des Anomalies du Développement (GAD) INSERM UMR1231, 2 Rue Angélique Ducoudray, F-21000 Dijon, France; 2 CHU Dijon Bourgogne, Laboratoire de Biologie de la Reproduction-CECOS, 14 rue Gaffarel, F-21000 Dijon, France

We read the study by Cannarella et al. [1] recently published in the *Journal of Clinical Medicine* with great interest. This systematic review and meta-analysis on the effects of assisted reproductive technologies (ARTs) on DNA methylation in human offspring could be of major importance for the health of children conceived through these techniques. Unfortunately, there are many limitations and confusing elements in this study that lead to questioning the veracity of the results.

It is first surprising that 50 articles were selected for quantitative synthesis (meta-analysis) (as shown in the first figure of the paper (page 4)), but only 12 were finally included. The methodology used in this systematic review to select studies and how they were distributed into the different analyses must be clarified.

The way the meta-analysis was conducted and its motivations are very confusing. The meta-analysis is greatly similar to the one published by Barberet et al. [2], but the number of studies included was higher in the study by Barberet et al. Cannarella et al. justified their approach to perform their meta-analysis by the fact that the study by Barberet et al. [2] was performed separately for each tissue “thus limiting the amount of data for each gene evaluated” (page 19) and then provided meta-analysis results by tissue but also grouped for all tissues. The choice to group the tissues together is not justified anywhere in the article with biological facts. However, this point has already been subject of debate for a previous meta-analysis [3,4], and there are very few arguments in favor of studying tissues together when assessing methylation. It is even more disturbing that the authors mention that their results could be limited by intra-tissue heterogeneity in placenta, but they do not discuss the inter-tissue heterogeneity. In addition, buccal smears studies were performed at childhood, whereas placenta and cord blood studies were performed at birth; thus, it is also highly questionable whether it is correct to pool data from different ages together, but this point is also not discussed.

The main result of this study suggests a hypomethylation in the *H19* CTCF3 region in ART newborns compared to controls. However, a duplication error was made in the analysis for buccal smears, and results from Puumala et al. [5] are wrong for spontaneous conceptions (*n* = 29, mean = 43.09, SD = 7.82). It is also hardly understandable that results from Choux et al. [6] are considered in placenta but are omitted in cord blood, whereas it was evaluated in the same study. The absence of two major studies on methylation is puzzling [7,8] and could be due to the omission of the term “methylation” in the search equation. Thus, as compared to the meta-analysis produced by Barberet et al. [2], the added value and quality of the reported findings have to be discussed herein.

There are also other figures in this study that seem to be wrong and may compromise the overall results for other genes. For *H19* CTCF6, the sample size is wrong for Sakian et al. [9] (nIVF = 39, nSC = 8), and we can wonder why ICSI samples from Wong et al. [10] and Sakian et al. [9] are not considered. In *KCNQ1OT1*, the sample size for Gomes et al. [11] is higher than the true value because the mean methylation corresponds to only three individuals with specific hypomethylation and the number of controls should be eight, as stated in Gomes et al. [11]: “In addition, umbilical cord and placenta samples were obtained from 8/30 and peripheral blood from 22/30 naturally conceived individuals (negative controls)” (page 472). Some formulations lack precision, particularly when referencing to “*KvDMR1* gene”, which is an imprinting region, or when referring to “*H19* methylation”, which does not allow us to know if the authors are discussing CTCF3, CTCF6, or *H19*/*IGF2* DMRs regions in each corresponding study.

We hope that these items can be corrected or justified and will no longer confound the results of Barberet et al. [2] who evaluated the same studies.
